# The Notification of Tuberculosis

**Published:** 1897-03-20

**Authors:** 


### The Notification of Tuberculosis.

Ever since the infective nature of tuberculosis was recognised the minds of many sanitarians have leaned towards making consumption a notifiable disease. The matter is one which has just now been brought to the front by what is going on in New York, where the Health Department has made the notification of all such cases compulsory on medical practitioners.

It is impossible to shut one's eyes to the fact that this is the direction in which many other sanitary authorities are now drifting, and that the notification and the State control of consumption may before long become burning questions.

Those, however, who have studied the causes of tuberculosis will recognise how shallow is the pathology of those who profess to be able to stop its ravages by notification, and other like measures for the muzzling of the microbe. The first, and the main, thing that has to be understood is, that consumption, when it permeates a community, is not merely the result of the scattering of the infection, but is the outcome of, and perhaps one might not unfitly call it the punishment for, certain well-known sanitary sins. We may, therefore, be allowed to doubt whether any sanitary administration will ever be able by aid of notification to absolve us from the consequences of our misdirected civilisation.

When we consider the amount of tuberculous meat which is being eaten, of milk which is being drunk, and of tuberculous dust which is being inhaled by people who never become tuberculous, and when we bear in mind the extent to which medical men, and still more to which nurses, are often exposed to infection without being attacked by the disease, the fact is forcibly borne in upon us that something more than the microbe is necessary to cause consumption.

What the contributory causes are is perfectly well known, and they may be summed up as being due to disease, to hereditary and social conditions, and to insanitary surroundings; and no one can observe the virulence of tuberculosis where these conditions prevail and its practical absence (even where there are plenty of bacilli) when these causes are not in operation, without becoming convinced that, as between the infection and what we have called the contributory conditions, the latter are by far the most important in the production of consumption.

Of the necessity of our municipal authorities undertaking a great crusade against tuberculosis we are convinced, but it must be undertaken in the right way. It is the suppression of the predisposing causes that we must aim at if we wish to see it lessening its hold upon the population; and it must be remembered that this indirect crusade against consumption was going on even before the discovery of the bacillus, and has been continued since with considerable effect.

Since 1875 immense strides have been made in improving the sanitary condition of the country, with the result that without the passage of a single law which could be looked upon as in any way tending to limit the distribution of the microbe, the prevalence of phthisis, which, after all, is the main tuberculous disease, and is the measure of the prevalence of all the rest, has very largely diminished, and it is in this direction that we must look for progress in the war against consumption.

When then we hear of proposals to render compulsory the notification of tuberculosis we cannot but ask, What will the sanitary authorities do with the notifications when they get them? If they are to be used as a means of getting at the patients, segregating them, which must practically mean imprisonment, or interfering with them in their occupations, taking away their means of earning their livelihood, and thus worrying them into hospitals, institutions, and workhouse infirmaries, we should be very sceptical both of the advantage and the justice of any such plan. If, however, notification is to be used as a means of finding out the localities in which phthisis prevails, and especially of the places in which it originates, and if the information so gained is to be made available to enforce the destruction of rookeries, the abolition of overcrowding, the prohibition of "sweating," and the general raising of the standard of comfort and of sanitation. then we may admit its usefulness. Unfortunately we cannot have much confidence in its being so used. It is plain to everyone that the knowledge we at present possess as to the existence of the contributory causes, to which the prevalence of consumption is so largely due, does not lead to their suppression. Our sanitary knowledge is, even now, far ahead of our sanitary performances, and when we see that these well recognised causes are allowed to continue, although their existence is a matter of common knowledge, we find it difficult to persuade ourselves that even notification would lead to their suppression. For municipal authorities to allow the building of rookeries and the shutting out of light and air and then to ask for notification of the tuberculosis so produced, seems absurd. It is not at present more knowledge that is wanted, but more willingness to act upon the knowledge we possess.

